# Supplementary material for: Identification of key odorants in honeysuckle by headspace-solid phase microextraction and solvent-assisted flavour evaporation with gas chromatography-mass spectrometry and gas chromatograph-olfactometry in combination with chemometrics
Source: PLoS One. 2020 Aug 20;15(8):e0237881. doi: 10.1371/journal.pone.0237881 (PMC7440650; doi:10.1371/journal.pone.0237881)
Supplement: S1 Table — (DOCX) [file pone.0237881.s001.docx]

**S1 Table.** Identification of volatile compounds in dry and brewed *Lonicera japonica Flos*, *Lonicera japonica Caulis*, *Lonicera Flos*, and *Lonicera Caulis* extracted using HS-SPME (80 °C for 30 min)

| **No.** | **Compounds** |  |  | **lonicera japonica** | | | | **Lonicera** | | | | **Identification**^§^ |
| --- | --- | --- | --- | --- | --- | --- | --- | --- | --- | --- | --- | --- |
|  |  | **Expt.**  **LRI**^†^ | **Ref.**  **LRI**^‡^ | **Flos** | | **Caulis** | | **Flos** | | **Caulis** | |  |
|  |  |  |  | **Dry** | **Brewed** | **Dry** | **Brewed** | **Dry** | **Brewed** | **Dry** | **Brewed** |  |
| 1 | Ethanol | 932 | 932 | - | - | 333786 ± 83539 | - | - | - | 270010 ± 15488 | - | MS, LRI, STD |
| 2 | 2-Ethyl furan^a^ | 957 | 950 | 338139 ± 12553 | 307687 ± 5716 | - | - | - | - | - | - | MS, LRI, STD |
| 3 | Pentanal^a^ | 965 | 979 | 2934735 ± 24066 | - | - | - | 971617 ± 64166 | 693299 ± 11428 | - | - | MS, LRI, STD |
| 4 | 2-Butenal | 1051 | 1047 | 43835326 ± 974238 | 10973821 ± 701037 | 196969 ± 2025 | 176244 ± 38504 | 806738 ± 71365 | 351616 ± 8468 | 645751 ± 69175 | 275668 ± 14405 | MS, LRI, STD |
| 5 | Hexanal^a,b,c^ | 1089 | 1083 | 3572869 ± 97938 | 103879 ± 1367 | 1184741 ± 272780 | 85209 ± 2381 | 1279629 ± 191802 | 1386449 ± 36063 | 1045333 ± 318422 | 1116411 ± 13222 | MS, LRI, STD |
| 6 | *trans*-2-Pentenal^a^ | 1137 | 1127 | 573342 ± 1613 | 354104 ± 8912 | - | - | - | 976404 ± 33873 | - | 316496 ± 14607 | MS, LRI, STD |
| 7 | 1-Penten-3-ol | 1155 | 1159 | 521038 ± 10575 | - | - | - | 381730 ± 40991 | 227967 ± 7860 | 103763 ± 14685 | - | MS, LRI, STD |
| 8 | Pyridine | 1188 | 1185 | - | - | 4449431 ± 112217 | 1407025 ± 92273 | - | - | 473071 ± 103140 | 382158 ± 701 | MS, LRI, STD |
| 9 | Heptanal^b^ | 1188 | 1184 | - | - | - | - | 153677 ± 3445 | 292232 ± 3460 | - | - | MS, LRI, STD |
| 10 | Methyl hexanoate | 1188 | 1184 | 1213899 ± 13192 | 548205 ± 18940 | - | - | - | - | - | - | MS, LRI, STD |
| 11 | Limonene^b^ | 1201 | 1200 | 1645762 ± 69521 | 386873 ± 22112 | 12125270 ± 156218 | 1022336 ± 36156 | 2685135 ± 52537 | 224250 ± 4677 | 7126862 ± 1724974 | 58194 ± 1532 | MS, LRI, STD |
| 12 | *trans*-2-Hexenal^b, d^ | 1224 | 1216 | 10450908 ± 179788 | 6179434 ± 1918931 | 305340 ± 88206 | - | 273193 ± 7744 | 547619 ± 10535 | 506771 ± 114750 | 433890 ± 7295 | MS, LRI, STD |
| 13 | 2-Pentyl furan^c,d^ | 1232 | 1231 | 205242 ± 2611 | 233703 ± 193 | 1318709 ± 2984 | 271311 ± 9619 | 162802 ± 8390 | 165236 ± 5294 | 325220 ± 87752 | 31838 ± 1342 | MS, LRI, STD |
| 14 | *γ*-Terpinene^b^ | 1241 | 1246 | - | - | - | - | - | 526850 ± 21154 | 276349 ± 83203 | - | MS, LRI, STD |
| 15 | Pentanol^a^ | 1245 | 1250 | 723026 ± 25235 | 295289 ± 18440 | 611013 ± 27582 | 266316 ± 15482 | 740298 ± 19336 | - | - | - | MS, LRI, STD |
| 16 | *β*-Ocimene^b^ | 1250 | 1250 | - | - | - | - | - | 153225 ± 4540 | - | - | MS, LRI, STD |
| 17 | 3-Octanone | 1256 | 1253 | 67444 ± 397 | 58985 ± 403 | 111461 ± 53490 | 204423 ± 3068 | - | - | - | - | MS, LRI |
| 18 | Styrene | 1258 | 1261 | - | - | 290275 ± 21104 | 127575 ± 4144 | - | - | 139372 ± 23504 | 36413 ± 1715 | MS, LRI |
| 19 | Methyl *trans*-3-hexenoate | 1259 | 1259 | 76865 ± 751 | 23175 ± 776 | - | - | - | - | - | - | MS, LRI |
| 20 | Hexyl acetate | 1269 | 1272 | 267963 ± 2833 | 94686 ± 1246 | - | - | - | - | - | - | MS, LRI, STD |
| 21 | *p*-Cymene^e^ | 1275 | 1272 | 238268 ± 10851 | - | 1384602 ± 15635 | 364877 ± 10350 | 548336 ± 9061 | 397526 ± 18434 | 258777 ± 80467 | 39157 ± 2643 | MS, LRI, STD |
| 22 | Terpinolene | 1284 | 1283 | - | - | - | - | - | 67490 ± 3149 | - | - | MS, LRI, STD |
| 23 | 2-Octanone | 1284 | 1287 | - | - | 207929 ± 37810 | 497861 ± 38628 | - | - | - | 37663 ± 1117 | MS, LRI, STD |
| 24 | Octanal^b^ | 1290 | 1289 | 124670 ± 3620 | - | 97082 ± 24424 | - | 337562 ± 7454 | 209204 ± 2332 | 203514 ± 60966 | 294909 ± 1857 | MS, LRI, STD |
| 25 | Acetoin | 1291 | 1284 | 1156518 ± 8339 | 1651934 ± 279327 | - | - | - | - | - | - | MS, LRI, STD |
| 26 | 1-Octen-3-one | 1301 | 1300 | 352789 ± 20154 | - | - | - | 172602 ± 7957 | - | - | 279464 ± 5444 | MS, LRI, STD |
| 27 | 1-Hydroxy-2-propanone | 1310 | 1300 | - | - | - | - | 799992 ± 14234 | - | - | - | MS, LRI |
| 28 | *cis*-2-Pentenol | 1316 | 1318 | - | - | - | - | 677380 ± 37353 | - | - | - | MS, LRI |
| 29 | 2-Heptanol^b^ | 1320 | 1320 | 340161 ± 11911 | 588441 ± 50575 | 37511 ± 4775 | 78935 ± 1183 | - | - | - | - | MS, LRI, STD |
| 30 | *trans*-2-Heptenal^a^ | 1330 | 1323 | 2013660 ± 82435 | 80044 ± 66270 | 290712 ± 257419 | - | 649658 ± 57585 | 790825 ± 25074 | 445995 ± 107179 | 818342 ± 11412 | MS, LRI, STD |
| 31 | 6-Methyl-5-hepten-2-one^b^ | 1339 | 1338 | 322577 ± 6158 | 354644 ± 28304 | 255321 ± 52011 | 279103 ± 13560 | 359608 ± 13639 | 549889 ± 23088 | 162305 ± 15253 | 241193 ± 2535 | MS, LRI, STD |
| 32 | Hexanol^b^ | 1346 | 1355 | 5729234 ± 247633 | 4587838 ± 196861 | 3553033 ± 45837 | 3021902 ± 187097 | 3386441 ± 55470 | 878073 ± 45451 | 349422 ± 73606 | 194571 ± 5377 | MS, LRI, STD |
| 33 | *trans*-3-Hexenol | 1359 | 1367 | 203578 ± 1434 | 95167 ± 4054 | - | - | - | - | - | - | MS, LRI, STD |
| 34 | *cis*-3-Hexenol^b^ | 1380 | 1382 | 10924011 ± 382708 | 4017676 ± 370018 | 142341 ± 2354 | 100155 ± 5536 | 607746 ± 19537 | 126864 ± 8860 | 151777 ± 35866 | 108963 ± 7343 | MS, LRI, STD |
| 35 | 2-Nonanone | 1387 | 1390 | - | - | - | 1416714 ± 678308 | - | - | - |  | MS, LRI, STD |
| 36 | Nonanal^a,b,d^ | 1394 | 1391 | 686248 ± 3152 | 37686 ± 1548 | 177386 ± 27238 | 51939 ± 2406 | 631252 ± 6561 | 485432 ± 10707 | 1051743 ± 400857 | 690481 ± 17005 | MS, LRI, STD |
| 37 | *trans*-2-Hexenol^b^ | 1399 | 1405 | 864539 ± 3746 | 1084998 ± 398706 | 64842 ± 1079 | 89915 ± 3485 | - | - | - | 81977 ± 3179 | MS, LRI, STD |
| 38 | *trans*, *trans*-2,4-Hexadienal^a^ | 1404 | 1400 | 458702 ± 5397 | 318594 ± 12654 | - | - | - | - | - |  | MS, LRI |
| 39 | 3-Octen-2-one | 1410 | 1411 | - | - | - | 701177 ± 94773 | 69988 ± 2055 | 193231 ± 10999 | 254821 ± 41110 | 318903 ± 3728 | MS, LRI |
| 40 | *trans*-2-Octenal | 1433 | 1429 | 295958 ± 1652 | 40591 ± 27337 | 166665 ± 83100 | - | 142978 ± 5579 | 479121 ± 15415 | 159104 ± 42357 | 407093 ± 6095 | MS, LRI, STD |
| 41 | 1-Octen-3-ol^a,d,e^ | 1441 | 1450 | 1076568 ± 2440 | 787085 ± 28725 | 805965 ± 65601 | 1169457 ± 42853 | 465557 ± 27729 | 751805 ± 36982 | 514064 ± 16311 | 659273 ± 4250 | MS, LRI, STD |
| 42 | Heptanol^b^ | 1448 | 1453 | 164914 ± 920 | 445821 ± 124633 | - | 370827 ± 15212 | 1051679 ± 36484 | 600651 ± 24924 | - | 98796 ± 2508 | MS, LRI, STD |
| 43 | Acetic acid^c^ | 1450 | 1449 | 18820361 ± 351666 | 154383 ± 27499 | 23936606 ± 395264 | 426058 ± 165957 | 7573116 ± 196902 | - | 4401589 ± 132864 | - | MS, LRI, STD |
| 44 | *α*-Cubebene | 1453 | 1463 | - | - | 1110500 ± 55571 | - | - | - | 150382 ± 49811 | - | MS, LRI |
| 45 | Furfural^a,c,d^ | 1469 | 1461 | 278934 ± 10820 | - | 523143 ± 2653 | - | 777781 ± 39779 | - | 248186 ± 63238 | - | MS, LRI, STD |
| 46 | 2-Ethyl hexanol | 1480 | 1491 | - | - |  | - | 86420 ± 1560 | - | 247242 ± 86648 | 395122 ± 4066 | MS, LRI, STD |
| 47 | 4-Vinyl pyridine^c^ | 1486 | 1486 | 547515 ± 10215 | 424600 ± 27212 | 546824 ± 3703 | 708532 ± 18475 | - | - | - | 199322 ± 2275 | MS, LRI |
| 48 | 2-Decanone | 1491 | 1494 | - | - | - | 61521 ± 17926 | - | - | - | - | MS, LRI |
| 49 | *α-*Copaene | 1491 | 1492 | - | - | 3004897 ± 235084 | - | - | - | 1104239 ± 158617 | - | MS, LRI |
| 50 | Decanal^b^ | 1498 | 1498 | 64419 ± 1970 | - | - | - | 125362 ± 3970 | 61434 ± 2754 | 436563 ± 149255 | 136159 ± 6498 | MS, LRI, STD |
| 51 | *trans*, *trans*-2,4-Heptadienal | 1501 | 1495 | 441421 ± 5452 | 863794 ± 356645 | - | - | 631866 ± 7565 | 3699132 ± 20563 | - | 568743 ± 4948 | MS, LRI, STD |
| 52 | 2-Nonanol | 1506 | 1521 | - | - | - | 90927 ± 2828 | - | - | - | - | MS, LRI |
| 53 | 3,5-Octadien-2-one^g^ | 1523 | 1522 | 659105 ± 4855 | 861421 ± 32821 | 284977 ± 28425 | 603766 ± 10215 | 484597 ± 26242 | 563380 ± 10584 | 395508 ± 57132 | 467730 ± 4771 | MS, LRI |
| 54 | Linalool^a,b,c,d,e.f^ | 1537 | 1547 | - | - | - | 302321 ± 7571 | - | 1750102 ± 58412 | - | - | MS, LRI, STD |
| 55 | Benzaldehyde^a,b,c,d^ | 1537 | 1520 | 3730574 ± 110448 | 2511316 ± 1073944 | 1988187 ± 159062 | - | 132185 ± 2376 | - | 864052 ± 281435 | 1669881 ± 10149 | MS, LRI, STD |
| 56 | Octanol^f^ | 1549 | 1557 | 2274686 ± 38718 | 1844165 ± 14840 | 525415 ± 40409 | 911466 ± 5682 | 5781711 ± 293975 | 3802795 ± 132625 | 418855 ± 103351 | 587467 ± 845 | MS, LRI, STD |
| 57 | 2,3-Butanediol | 1570 | 1565 | 3365719 ± 282120 | - | - | - | - | - | - | - | MS, LRI, STD |
| 58 | 5-Methyl furfural | 1583 | 1570 | - | - | - | - | - | 97929 ± 1439 | - | - | MS, LRI, STD |
| 59 | 6-Methyl-3,5-heptadiene-2-one | 1599 | 1602 | - | - | - | 192822 ± 6847 | - | 107897 ± 6610 | - | 118493 ± 1415 | MS, LRI, STD |
| 60 | *β*-Caryophyllene ^a,b,d,e^ | 1600 | 1595 | - | - | 693033 ± 27076 | - | - | - | 715751 ± 416256 | - | MS, LRI, STD |
| 61 | 4-Terpineol^c^ | 1604 | 1602 | - | - | - | 432512 ± 22485 | 123516 ± 3003 | 860940 ± 8625 | - | 128967 ± 3618 | MS, LRI, STD |
| 62 | *trans*-2-Octenol | 1605 | 1614 | 405924 ± 4443 | - | - | - | - | 550950 ± 35460 | - | - | MS, LRI |
| 63 | Butanoic acid | 1619 | 1625 | - | - | 141413 ± 52715 | - | 152173 ± 65826 | - | - | - | MS, LRI, STD |
| 64 | Methyl benzoate^b^ | 1627 | 1612 | 257766 ± 2838 | 590355 ± 14877 | - | - | - |  | - | - | MS, LRI, STD |
| 65 | *γ*-Butanolactone | 1647 | 1632 | 3570670 ± 57436 | - | 3254003 ± 13734 | - | - | - | - | - | MS, LRI, STD |
| 66 | 1-Nonanol | 1646 | 1660 | - | 194279 ± 20933 | - | 558436 ± 25162 | 1053592 ± 22803 | 383436 ± 19083 |  | 181880 ± 8162 | MS, LRI, STD |
| 67 | 3-Methyl butanoic acid | 1662 | 1666 | 1507712 ± 7133 | - | - | - | - | - | - | - | MS, LRI, STD |
| 68 | 2-Hydroxy benzaldehyde | 1672 | 1672 | - | - | - | 348314 ± 39674 | - | - | - | - | MS, LRI |
| 69 | *α-*Humulene | 1676 | 1667 | - | - | 636583 ± 24096 | - | - | - | 416654 ± 344952 | - | MS, LRI, STD |
| 70 | Neral^b^ | 1684 | 1680 | - | 52983 ± 6157 | - | - | - | - | - | - | MS, LRI, STD |
| 71 | *γ*-Muurolene | 1691 | 1692 | - | - | 2966362 ± 166086 | - | - | - | 478921 ± 125865 | - | MS, LRI, STD |
| 72 | *α-*Terpineol^b,f^ | 1696 | 1697 | 114197 ± 4926 | - | - | - | - | 208216 ± 5474 | - | - | MS, LRI, STD |
| 73 | *γ*-Hexanolactone | 1723 | 1694 | 160717 ± 1017 | - | - | - | - | - | - | - | MS, LRI, STD |
| 74 | α-Muurolene | 1725 | 1726 | - | - | 586575 ± 33754 | - | - | - | 454172 ± 95272 | - | MS, LRI, STD |
| 75 | Pentanoic acid | 1731 | 1733 | 560167 ± 11944 | - | 1316043 ± 269165 | - | 157256 ± 31256 | - | - | - | MS, LRI, STD |
| 76 | *trans*-Linalool 3,7-oxide^c^ | 1739 | 1739 |  | - |  |  | - | 168934 ± 8419 | 385853 ± 101427 | 348839 ± 3553 | MS, LRI |
| 77 | Geranial^b^ | 1739 | 1732 | 76147 ± 541 | 194842 ± 6469 | - | - | - | - | - | - | MS, LRI, STD |
| 78 | Citronellol^b^ | 1754 | 1765 | - | 56898 ± 1606 | - | - | - | - | - | - | MS, LRI, STD |
| 79 | Decanol | 1760 | 1760 | 45874 ± 1398 | 27136 ± 3057 | 81763 ± 39177 | - | 3177944 ± 177702 | 1485363 ± 43923 | 131486 ± 37829 | - | MS, LRI, STD |
| 80 | Methyl phenylacetate^f^ | 1761 | 1750 | - | 33930 ± 848 | - | - | - | - | - | - | MS, LRI, STD |
| 81 | Geraniol^b,e,f^ | 1839 | 1847 | 115142 ± 795 | 66922 ± 19003 | - | - | 87684 ± 3378 | 155633 ± 4923 | - | 60141 ± 3283 | MS, LRI, STD |
| 82 | Hexanoic acid^d^ | 1840 | 1846 | 5604834 ± 33255 | 815549 ± 61127 | 9116948 ± 1520614 | - | 1128835 ± 34829 | - | 737612 ± 143577 | 125884 ± 2295 | MS, LRI, STD |
| 83 | 2-Methoxy phenol | 1864 | 1861 | - | - | - | 15666845 ± 331314 | - | - | - | - | MS, LRI, STD |
| 84 | Benzyl alcohol^b,e^ | 1879 | 1870 | 2749982 ± 77545 | 926949 ± 107781 | 380216 ± 32088 | 336929 ± 1477 | 367005 ± 12703 | 111184 ± 3113 | 213151 ± 57423 | 174722 ± 4095 | MS, LRI, STD |
| 85 | 2-Phenylethyl alcohol^a,b,c,d,e^ | 1914 | 1906 | 2254451 ± 115239 | 1060042 ± 48161 | 287851 ± 17657 | 438449 ± 15427 | 6163234 ± 217974 | 1247572 ± 14986 | 145533 ± 23181 | 93758 ± 3937 | MS, LRI, STD |
| 86 | Heptanoic acid^d^ | 1945 | 1950 | 198736 ± 73682 | - | 360703 ± 70635 | 328133 ± 17372 | 228198 ± 25248 | - | 186470 ± 74052 | - | MS, LRI, STD |
| 87 | 3-Hexenoic acid | 1955 | 1954 | 569591 ± 17208 | - | - | - | - | - | - | - | MS, LRI, STD |
| 88 | *cis*-Jasmone^b,e,f^ | 1956 | 1961 | - | 22826 ± 1354 | - | - | - | - | - | - | MS, LRI |
| 89 | *trans*-2-Hexenoic acid | 1964 | 1967 | 778309 ± 28480 | 78237 ± 4386 | - | - | - | - | - | - | MS, LRI, STD |
| 90 | *β*-Ionone^d^ | 1971 | 1971 | 202647 ± 5969 | 246184 ± 10724 | 131583 ± 1883 | 105144 ± 2912 | 224267 ± 14272 | 172171 ± 674 |  | 93676 ± 3351 | MS, LRI, STD |
| 91 | Phenol | 2004 | 2000 | 193684 ± 1400 | 64828 ± 1132 | 106476 ± 7868 | 92330 ± 11808 | 202156 ± 9724 | 139356 ± 1721 | 48788 ± 2533 | - | MS, LRI, STD |
| 92 | 2-Methyl phenol | 2011 | 2008 | 26184 ± 437 | - | - | - | - | - | - | - | MS, LRI, STD |
| 93 | *γ-*Nonanolactone | 2041 | 2024 | - | - | 161514 ± 11628 | 466456 ± 7593 | - | - | - | - | MS, LRI, STD |
| 94 | Octanoic acid^d^ | 2052 | 2060 | 571129 ± 195107 | 203092 ± 57929 | 916411 ± 3882 | 491388 ± 36813 | 668046 ± 44823 | 36154 ± 1495 | - | 157466 ± 19323 | MS, LRI, STD |
| 95 | Nonanoic acid^d^ | 2157 | 2171 | 104483 ± 91350 | 202103 ± 139375 | 489701 ± 88337 | 373207 ± 49709 | 507997 ± 217272 | 63173 ± 14420 | 370546 ± 421360 | 287204 ± 75356 | MS, LRI, STD |
| 96 | Eugenol^c,d,e,f^ | 2169 | 2169 | 24758 ± 2923 | 62772 ± 4156 | - | 68354 ± 1489 | - |  | - | 81080 ± 565 | MS, LRI, STD |
| 97 | 2-Methoxy-4-vinylphenol | 2197 | 2188 | - | 365219 ± 219127 | 74029 ± 10019 | 2472505 ± 131402 | - | 41376 ± 2369 | - | - | MS, LRI, STD |
| 98 | Methyl hexadecanoate | 2206 | 2208 | 156349 ± 7394 | - | 43356 ± 499 | - | - | - | - | - | MS, LRI |
| 99 | Isophytol | 2297 | 2296 | 85950 ± 4293 | - | - | - | - | - | - | - | MS, LRI |
| 100 | Dihydroactinidiolide | 2377 | 2331 | 146027 ± 3275 | 67582 ± 5722 | 79446 ± 251 | - | - | - | - | - | MS, LRI, STD |
| 101 | Benzoic acid^b^ | 2445 | 2412 | 249370 ± 9262 | - | 22742 ± 116 | - | - | - | - | - | MS, LRI, STD |
| 102 | Vanillin | 2578 | 2568 | - | - | 44643 ± 237 | - | - | - | - | - | MS, LRI, STD |

The data is average peak area of each compound ± its standard deviation of triplicate analysis.

“-” means compounds were not detected.

^†^Expt. LRI: linear retention index on an HP-Innowax column relative to C7-C40 alkane standards.

^‡^Ref. LRI: Reference retention index values from literature: NIST 14 MS library

^§^Identification methods: MS = Comparison with mass spectrum of the compound in the NIST library version 2.2; LRI = Comparison of retention index with that of the compound reported in cited literature(s) or in the NIST 14.0 library version 2.2; STD= Comparison of experimental to standards retention indices.

^a^ Compounds reported in Ref. [27]

^b^ Compounds reported in Ref. [28]

^c^ Compounds reported in Ref. [30]

^d^ Compounds reported in Ref. [33]

^e^ Compounds reported in Ref. [32]

^f^ Compounds reported in Ref. [31]
