# Supplementary material for: Identification of key odorants in honeysuckle by headspace-solid phase microextraction and solvent-assisted flavour evaporation with gas chromatography-mass spectrometry and gas chromatograph-olfactometry in combination with chemometrics
Source: PLoS One. 2020 Aug 20;15(8):e0237881. doi: 10.1371/journal.pone.0237881 (PMC7440650; doi:10.1371/journal.pone.0237881)
Supplement: S2 Table — (DOCX) [file pone.0237881.s002.docx]

**S2 Table.** Identification of volatile compounds and concentration (ng/mL) in *Lonicera japonica Flos*, *Lonicera Flos*, *Lonicera japonica Caulis*, and *Lonicera Caulis* extracted using SAFE

| **No.** | **Compounds** | **Expt. LRI**^†^ | **Ref. LRI**^‡^ | **Lonicera japonica Flos** | **Lonicera Flos** | **Lonicera japonica Caulis** | **Lonicera Caulis** | **Identification**^§^ |
| --- | --- | --- | --- | --- | --- | --- | --- | --- |
| 1 | 1-Penten-3-one | 1031 | 1019 | 23.51 ± 0.9 | 19.41 ± 1.15 | - | - | MS, LRI |
| 2 | 2-Butenal | 1051 | 1047 | 1236.37 ± 112.84 | 23.41 ± 0.72 | 18.75 ± 0.52 | 14.58 ± 0.81 | MS, LRI, STD |
| 3 | Hexanal^a,b^ | 1089 | 1083 | 139.45 ± 8.86 | 98.08 ± 3.51 | 98.74 ± 2.58 | 42.85 ± 1.8 | MS, LRI, STD |
| 4 | 2-Methyl-2-butenal | 1104 | 1095 | 6.97 ± 0.04 | 2.13 ± 0.07 | - | - | MS, LRI |
| 5 | *trans*-2-Pentenal^a^ | 1137 | 1127 | 34.21 ± 2.69 | 76.32 ± 3.26 | - | - | MS, LRI, STD |
| 6 | Butanol | 1141 | 1142 | - | 30.21 ± 1.57 | 7.33 ± 0.46 | 25.13 ± 0.54 | MS, LRI, STD |
| 7 | 1-Penten-3-ol | 1155 | 1159 | - | 190.94 ± 4.54 | 13.72 ± 0.2 | 19.18 ± 0.25 | MS, LRI, STD |
| 8 | 2-Heptanone | 1184 | 1182 | - | 1.26 ± 0.02 | 5.75 ± 0.1 | - | MS, LRI, STD |
| 9 | Heptanal^b^ | 1188 | 1184 | - | 4.86 ± 0.22 | - | - | MS, LRI, STD |
| 10 | Pyridine | 1188 | 1185 | - | - | 98.02 ± 5.37 | 13.15 ± 0.61 | MS, LRI, STD |
| 11 | Methyl hexanoate | 1188 | 1184 | 18.72 ± 1.04 | - | - | - | MS, LRI, STD |
| 12 | 3-Methyl-2-butenal | 1202 | 1215 | - | 4.14 ± 0.14 | - | - | MS, LRI, STD |
| 13 | 3-Methyl butanol | 1202 | 1209 | 80.2 ± 5.6 | - | 153.2 ± 2.17 | 5.81 ± 0.34 | MS, LRI |
| 14 | Eucalyptol | 1210 | 1213 | - | - | 4.26 ± 0.39 |  | MS, LRI, STD |
| 15 | *trans*-2-Hexenal^b,d^ | 1224 | 1216 | 491.46 ± 34.74 | 20.55 ± 0.9 | 12.89 ± 0.19 | 14.75 ± 1.15 | MS, LRI, STD |
| 16 | Pentanol^a^ | 1245 | 1250 | 53.4 ± 1.62 | 70.19 ± 2.37 | 55.54 ± 0.27 | 15.77 ± 0.5 | MS, LRI, STD |
| 17 | 3-Octanone | 1256 | 1253 | - | - | 1.4 ± 0.06 | - | MS, LRI |
| 18 | 2-Methyl pyrazine | 1290 | 1266 | - | 3.53 ± 0.13 | 4.19 ± 0.05 | - | MS, LRI, STD |
| 19 | Acetoin | 1291 | 1284 | 95.13 ± 7.6 | 7.94 ± 0.08 | - | - | MS, LRI, STD |
| 20 | 2,3-Methyl butanol | 1292 | 1291 | - | - | 6.97 ± 0.36 | - | MS, LRI |
| 21 | 3-Methyl pyridine | 1292 | 1292 | 1.36 ± 0.04 | 0.77 ± 0.04 | 2.56 ± 0.19 | 5.18 ± 0.52 | MS, LRI, STD |
| 22 | *trans*-2-Heptenal^a^ | 1330 | 1323 | 11.82 ± 0.51 | 14.54 ± 0.41 | - | - | MS, LRI, STD |
| 23 | 2,6-Dimethyl pyrazine | 1333 | 1328 | - | 2.01 ± 0.09 | 1.68 ± 0.03 | 1.43 ± 0.03 | MS, LRI, STD |
| 24 | 6-Methyl-5-hepten-2-one^b^ | 1339 | 1338 | 8.48 ± 0.43 | 12.46 ± 0.37 | 9.91 ± 0.41 | 4.95 ± 0.16 | MS, LRI |
| 25 | Hexanol^b^ | 1346 | 1355 | 268.17 ± 14.15 | 97.89 ± 2.14 | 233.68 ± 2.62 | 11.55 ± 1.03 | MS, LRI, STD |
| 26 | *trans*-3-Hexenol | 1359 | 1367 | 10.44 ± 0.13 | - | - | - | MS, LRI, STD |
| 27 | *cis*-3-Hexenol^b^ | 1380 | 1382 | 520.63 ± 22.57 | 23.66 ± 1.64 | 12.51 ± 0.57 | 8.37 ± 0.4 | MS, LRI, STD |
| 28 | 4-Methyl-3-pentenol | 1383 | 1385 | - | 19.51 ± 0.65 | - | 4.03 ± 0.27 | MS, LRI |
| 29 | 2-Ethyl-5-methyl pyrazine | 1388 | 1387 | - | 1.55 ± 0.01 | - | - | MS, LRI, STD |
| 30 | 3-Ethyl pyridine | 1390 | 1377 | 2.99 ± 0.23 | - | 5.39 ± 1.26 | - | MS, LRI, STD |
| 31 | Nonanal^a,b,d^ | 1394 | 1391 | 3.2 ± 0.15 | 2.37 ± 0.06 | - | 4.62 ± 0.42 | MS, LRI, STD |
| 32 | *trans*-2-Hexenol^b^ | 1399 | 1405 | 35.45 ± 2.07 | 0.97 ± 0.08 | 5.32 ± 0.26 | 2.69 ± 0.47 | MS, LRI, STD |
| 33 | *trans*,*trans*-2,4-Hexadienal^a^ | 1404 | 1400 | 3.02 ± 0.09 | - | - | - | MS, LRI |
| 34 | 3-Octen-2-one | 1410 | 1411 | - | - | 17.52 ± 0.14 | 8.98 ± 0.6 | MS, LRI |
| 35 | *cis*-2-Hexenol | 1416 | 1416 | 2.84 ± 0.26 | - | - | - | MS, LRI |
| 36 | *trans*-2-Octenal | 1433 | 1429 | - | 9.7 ± 0.32 | 7.82 ± 0.21 | 9.14 ± 0.66 | MS, LRI, STD |
| 37 | 1-Octen-3-ol^b,d,e^ | 1441 | 1450 | 17.42 ± 0.51 | 12.43 ± 0.85 | 26.32 ± 0.81 | 12.48 ± 0.35 | MS, LRI, STD |
| 38 | Heptanol^b^ | 1448 | 1453 | 5.24 ± 0.72 | 16.19 ± 1.12 | 9.34 ± 0.92 | - | MS, LRI, STD |
| 39 | Acetic acid^f^ | 1450 | 1449 | 17.39 ± 2.75 | 2.07 ± 0.22 | 11.36 ± 3.49 | - | MS, LRI, STD |
| 40 | Furfural^a,d,f^ | 1469 | 1461 | - | - | 17.1 ± 0.14 | - | MS, LRI, STD |
| 41 | 2-Ethyl-hexanol | 1480 | 1491 | - | 5.03 ± 0.15 | 8.89 ± 0.35 | 11.48 ± 0.92 | MS, LRI, STD |
| 42 | 4-Vinyl pyridine^f^ | 1486 | 1486 | - | - | 30.64 ± 0.17 | 7.97 ± 0.62 | MS, LRI |
| 43 | 2-Methyl-6-vinyl pyrazine | 1495 | 1490 | - | 5.04 ± 0.23 | - | - | MS, LRI |
| 44 | *trans*,*trans*-2,4-Heptadienal | 1501 | 1495 | 75.23 ± 4.2 | 183.27 ± 6.02 | 7.43 ± 0.29 | 16.88 ± 0.59 | MS, LRI, STD |
| 45 | 1-(2-Furanyl)-ethanone | 1512 | 1499 | - | 1.31 ± 0.07 | - | - | MS, LRI, STD |
| 46 | 2,4,6-Octatrienal | 1530 | 1530 | 2.13 ± 0.08 | - | - | - | MS, LRI |
| 47 | Benzaldehyde^a,b,d,f^ | 1537 | 1520 | 132.09 ± 9.54 | 17.05 ± 1.03 | 77.13 ± 3.78 | 38.27 ± 1.19 | MS, LRI, STD |
| 48 | Linalool^a,b,c,d,e.f^ | 1537 | 1547 | - | 16.92 ± 1.08 | - | - | MS, LRI, STD |
| 49 | Octanol^b^ | 1549 | 1557 | 28.15 ± 2.7 | 69.84 ± 2.62 | 13.42 ± 0.64 | 10.44 ± 1.15 | MS, LRI, STD |
| 50 | *trans*,*trans*-3,5-Octadien-2-one | 1577 | 1570 | 31.16 ± 1.89 | 38.92 ± 0.56 | 16.23 ± 0.53 | 7.11 ± 0.45 | MS, LRI |
| 51 | 5-Methyl furfural | 1583 | 1570 | 1.52 ± 0.06 | 4.81 ± 0.1 | - | - | MS, LRI, STD |
| 52 | *trans*,*cis*-2,6-Nonadienal | 1584 | 1584 | - | - | 2.93 ± 0.24 | - | MS, LRI, STD |
| 53 | *trans*,*trans*-2,4-Octadienal | 1597 | 1597 | - | 3.46 ± 0.18 | - | - | MS, LRI |
| 54 | 6-Methyl-3,5-heptadien-2-one | 1599 | 1602 | - | 3.18 ± 0.06 | 6.8 ± 0.23 | 3.66 ± 0.23 | MS, LRI, STD |
| 55 | 4-Terpineol | 1604 | 1602 | - | - | 9.02 ± 0.34 | 9.09 ± 2.81 | MS, LRI, STD |
| 56 | *γ-*Pentalactone | 1626 | 1616 | - | - | 30.25 ± 1.17 | 8.17 ± 0.37 | MS, LRI, STD |
| 57 | Methyl benzoate^b^ | 1631 | 1621 | 18.09 ± 1.32 | - | - | - | MS, LRI, STD |
| 58 | 1-Methyl-2-formyl pyrrole | 1633 | 1626 | - | 1.65 ± 0.09 | - | - | MS, LRI |
| 59 | 2-Methyl benzaldehyde | 1636 | 1632 | - | - | 2.92 ± 0.33 |  | MS, LRI |
| 60 | *γ-*Butanolactone | 1637 | 1632 | 213.29 ± 16.99 | - | 260.12 ± 14.59 | 17.54 ± 0.93 | MS, LRI, STD |
| 61 | Phenylacetaldehyde | 1640 | 1640 | - | - | 29.05 ± 1.91 | 8.32 ± 0.65 | MS, LRI, STD |
| 62 | Acetophenone | 1647 | 1647 | - | - | - | 4.39 ± 0.37 | MS, LRI, STD |
| 63 | 1-Nonanol | 1646 | 1660 | - | 23.32 ± 0.22 | - | - | MS, LRI, STD |
| 64 | 2-Furanmethanol | 1660 | 1660 | - | 13.66 ± 0.73 | - | - | MS, LRI, STD |
| 65 | 3-Methyl butanoic acid | 1662 | 1666 | 88.77 ± 12.58 | - | - | - | MS, LRI, STD |
| 66 | 2-Hydroxy benzaldehyde | 1672 | 1672 | - | - | 20.22 ± 2.04 | - | MS, LRI |
| 67 | Neral^b^ | 1684 | 1680 | - | - | - | 7.39 ± 2.34 | MS, LRI, STD |
| 68 | *cis*-3-Nonen-1-ol | 1682 | 1682 | 11.97 ± 0.24 | - | - | - | MS, LRI |
| 69 | *α*-Terpineol^b,c^ | 1696 | 1697 | 6.82 ± 0.56 | 5.82 ± 0.1 | - | 7.39 ± 1.35 | MS, LRI, STD |
| 70 | *γ*-Hexanolactone | 1723 | 1694 | 11.76 ± 0.87 | 1.83 ± 0.02 | 27.71 ± 1.65 | 2.45 ± 0.07 | MS, LRI, STD |
| 71 | Pentanoic acid | 1731 | 1733 | 12.06 ± 2.68 | - | 22.83 ± 1.4 |  | MS, LRI, STD |
| 72 | *trans*-Linalool 3,7-oxide^b^ | 1739 | 1739 | - | - | - | 18.44 ± 1.23 | MS, LRI |
| 73 | Decanol | 1749 | 1760 | - | 21.92 ± 0.8 | - | - | MS, LRI, STD |
| 74 | Methyl salicylate | 1789 | 1765 | - | - | 4.39 ± 0.1 | - | MS, LRI, STD |
| 75 | 1-Phenethyl alcohol | 1801 | 1801 | 1.83 ± 0.02 | - | 2.55 ± 0.23 | - | MS, LRI, STD |
| 76 | *trans*,*trans*-2,4-Decadienal | 1817 | 1811 | 4.7 ± 0.1 | 2.88 ± 0.16 | - | - | MS, LRI, STD |
| 77 | 2-Phenethyl acetate | 1820 | 1813 | - |  | - | - | MS, LRI, STD |
| 78 | Geraniol^b,c,e^ | 1839 | 1847 | - | 13.51 ± 1.24 | - | - | MS, LRI, STD |
| 79 | Hexanoic acid^d^ | 1840 | 1846 | 228.68 ± 20.04 | - | 445.66 ± 15.53 | 18.48 ± 2.28 | MS, LRI, STD |
| 80 | *trans*-Geranylacetone^b,c^ | 1854 | 1859 | 3.83 ± 0.26 | 2.76 ± 0.12 | - | 2.46 ± 0.14 | MS, LRI, STD |
| 81 | 2-Methoxy-phenol | 1864 | 1861 | - | - | 12.17 ± 0.58 | - | MS, LRI, STD |
| 82 | Benzyl alcohol^b,e^ | 1879 | 1870 | 177.31 ± 12.28 | 16.23 ± 0.23 | 35.26 ± 0.24 | 15.79 ± 0.71 | MS, LRI, STD |
| 83 | 2-Phenylethyl alcohol^a,b,d,e,f^ | 1914 | 1906 | 182.33 ± 9.65 | 124.03 ± 6.34 | 32.07 ± 2.84 | 10.17 ± 0.25 | MS, LRI, STD |
| 84 | *γ*-Octalactone | 1930 | 1910 | - | - | 3.25 ± 0.16 | - | MS, LRI, STD |
| 85 | *trans*-3-Hexenoic acid | 1953 | 1930 | 21.14 ± 3.66 | - | - | - | MS, LRI |
| 86 | Heptanoic acid^d^ | 1945 | 1950 | - | - | 33.13 ± 2.85 | - | MS, LRI, STD |
| 87 | *trans*-2-Hexenoic acid | 1964 | 1967 | 49.92 ± 5.83 | - | - | - | MS, LRI, STD |
| 88 | *β*-Ionone^d^ | 1971 | 1971 | 15.88 ± 2.1 | 3.89 ± 0.19 | 4.22 ± 0.05 | 4.02 ± 0.48 | MS, LRI, STD |
| 89 | 2-Acetyl pyrrole | 1973 | 1973 | 9.31 ± 0.77 | 17.3 ± 0.32 | 14.86 ± 0.47 | 3.04 ± 0.14 | MS, LRI, STD |
| 90 | Phenol | 2004 | 2000 | 11.29 ± 0.19 | 9.49 ± 0.78 | 6.52 ± 0.21 | 2.97 ± 0.08 | MS, LRI, STD |
| 91 | Methyl eugenol | 2009 | 2013 | - | - | - | 8.19 ± 0.07 | MS, LRI, STD |
| 92 | Pyrrole-2-carboxaldehyde | 2033 | 2030 | 2.8 ± 0.09 | 5.53 ± 0.3 | 6.17 ± 0.12 | - | MS, LRI |
| 93 | *γ*-Nonalactone | 2041 | 2024 | 2.33 ± 0.18 | - | 34.95 ± 2.39 | 2.44 ± 0.17 | MS, LRI, STD |
| 94 | Octanoic acid^d^ | 2052 | 2060 | 12.43 ± 1.96 | 1.36 ± 0.08 | 41.02 ± 2.12 | 4.82 ± 0.39 | MS, LRI, STD |
| 95 | *p*-Cresol | 2080 | 2080 | - | 1.86 ± 0.11 | - | - | MS, LRI, STD |
| 96 | *m*-Cresol | 2087 | 2091 | - | 7.15 ± 0.45 | - | - | MS, LRI, STD |
| 97 | Nonanoic acid^d^ | 2157 | 2171 | 8.7 ± 0.7 | 2.48 ± 0.07 | 51.52 ± 3.21 | 25.86 ± 1.6 | MS, LRI, STD |
| 98 | Eugenol^b,d,e,f,^ | 2169 | 2169 | 6.31 ± 0.35 | 2.3 ± 0.12 | 6.47 ± 0.15 | 5.1 ± 1.04 | MS, LRI, STD |
| 99 | 4-Ethyl phenol | 2179 | 2187 | 0.88 ± 0.02 | 1.05 ± 0.05 | - | 4.07 ± 0.32 | MS, LRI, STD |
| 100 | 2-Methoxy-4-vinyl phenol | 2197 | 2188 | - | 2.79 ± 1.34 | 3.92 ± 0.18 | 2.06 ± 0.19 | MS, LRI, STD |
| 101 | Piperonal | 2248 | 2212 | - | 1.31 ± 0.06 | - | - | MS, LRI, STD |
| 102 | Decanoic acid | 2262 | 2276 | 5.42 ± 0.44 | - | 31.07 ± 0.72 | 17.19 ± 1.1 | MS, LRI, STD |
| 103 | Geranic acid | 2329 | 2347 | - | 1.91 ± 0.06 | - | - | MS, LRI |
| 104 | Dihydroactinidiolide | 2377 | 2331 | - | - | 15.19 ± 0.33 | - | MS, LRI, STD |
| 105 | Benzoic acid^b^ | 2445 | 2412 | 20.22 ± 5.18 |  | - | - | MS, LRI, STD |
| 106 | Indole | 2455 | 2445 | - | 1.48 ± 0.04 | - | - | MS, LRI, STD |
| 107 | Dodecanoic acid | 2480 | 2498 | 12.03 ± 1.47 | - | - | - | MS, LRI, STD |
| 108 | Vanillin | 2578 | 2568 | 9.12 ± 0.8 | 13.27 ± 0.68 | 61.53 ± 4.14 | 16.37 ± 4.92 | MS, LRI, STD |

The data is average concentration of each compound± its standard deviation of triplicate analysis.

“-” means compounds were not detected.

^†^Expt. LRI: linear retention index on an HP-Innowax column relative to C7-C40 alkane standards.

^‡^Ref. LRI: Reference retention index values from literature: NIST 14 MS library

^§^Identification methods: MS = Comparison with mass spectrum of the compound in the NIST library version 2.2; LRI = Comparison of retention index with that of the compound reported in cited literature(s) or in the NIST 14.0 library version 2.2; STD= Comparison of experimental to standards retention indices.

^a^ Compounds reported in Ref. [27]

^b^ Compounds reported in Ref. [28]

^c^ Compounds reported in Ref. [30]

^d^ Compounds reported in Ref. [33]

^e^ Compounds reported in Ref. [32]

^f^ Compounds reported in Ref. [31]
